# The Implementation Playbook: study protocol for the development and feasibility evaluation of a digital tool for effective implementation of evidence-based innovations

**DOI:** 10.1186/s43058-023-00402-w

**Published:** 2023-03-07

**Authors:** Melanie Barwick, Jacquie Brown, Kadia Petricca, Bonnie Stevens, Byron J. Powell, Alexia Jaouich, Jill Shakespeare, Emily Seto

**Affiliations:** 1 Research Institute, The Hospital for Sick Children, Toronto, Canada; 2 Department of Psychiatry, University of Toronto, Toronto, Canada; 3 Institute of Health Policy, Management and Evaluation, Dalla Lana School of Public Health, University of Toronto, Toronto, Canada; 4 Social and Behavioural Health Sciences, Dalla Lana School of Public Health, University of Toronto, Toronto, Canada; 5 Jacquie Brown and Associates, Toronto, Canada; 6 Lawrence S Bloomberg Faculty of Nursing, University of Toronto, Toronto, Canada; 7 Center for Mental Health Services Research, Brown School, Washington University in St Louis, St. Louis, MO USA; 8 Division of Infectious Diseases, John T. Milliken Department of Medicine, School of Medicine, Washington University in St. Louis, St. Louis, MO USA; 9 Center for Dissemination & Implementation, Institute for Public Health, Washington University in St. Louis, St. Louis, MO, USA; 10 Stepped Care Solutions, Mount Pearl, Newfoundland, Canada; 11 Provincial System Support Program, Centre for Addiction and Mental Health, Toronto, Canada; 12 Centre for Digital Therapeutics, Techna Institute, University Health Network, Toronto, Canada

**Keywords:** eHealth technology, Implementation science, Implementation facilitation, Digital tools

## Abstract

**Background:**

Evidence-based innovations can improve health outcomes, but only if successfully implemented. Implementation can be complex, highly susceptible to failure, costly and resource intensive. Internationally, there is an urgent need to improve the implementation of effective innovations. Successful implementation is best guided by implementation science, but organizations lack implementation know-how and have difficulty applying it. Implementation support is typically shared in static, non-interactive, overly academic guides and is rarely evaluated. In-person implementation facilitation is often soft-funded, costly, and scarce. This study seeks to improve effective implementation by (1) developing a first-in-kind digital tool to guide pragmatic, empirically based and self-directed implementation planning in real-time; and (2) exploring the tool’s feasibility in six health organizations implementing different innovations.

**Methods:**

Ideation emerged from a paper-based resource, The Implementation Game©, and a revision called The Implementation Roadmap©; both integrate core implementation components from evidence, models and frameworks to guide structured, explicit, and pragmatic planning. Prior funding also generated user personas and high-level product requirements. This study will design, develop, and evaluate the feasibility of a digital tool called The Implementation Playbook©. In Phase 1, user-centred design and usability testing will inform tool content, visual interface, and functions to produce a minimum viable product. Phase 2 will explore the Playbook’s feasibility in six purposefully selected health organizations sampled for maximum variation. Organizations will use the Playbook for up to 24 months to implement an innovation of their choosing. Mixed methods will gather: (i) field notes from implementation team check-in meetings; (ii) interviews with implementation teams about their experience using the tool; (iii) user free-form content entered into the tool as teams work through implementation planning; (iv) Organizational Readiness for Implementing Change questionnaire; (v) System Usability Scale; and (vi) tool metrics on how users progressed through activities and the time required to do so.

**Discussion:**

Effective implementation of evidence-based innovations is essential for optimal health. We seek to develop a prototype digital tool and demonstrate its feasibility and usefulness across organizations implementing different innovations. This technology could fill a significant need globally, be highly scalable, and potentially valid for diverse organizations implementing various innovations.

Contributions to the literature

- The Implementation Playbook (TIP) integrates key implementation evidence, models, and frameworks in a novel and pragmatic way to provide a first-in-kind digital tool that will enable organizations to implement innovations in a self-directed manner guided by implementation science.
- Feasibility testing in six health organizations will inform whether TIP is feasible to use and can enable self-directed implementation across various contexts and innovations.
- This study will advance our understanding of the feasibility of self-directed implementation using a first-in-kind digital tool and whether an effectiveness study is suitable.

## Background

Evidence-based innovations (EBIs) are clinical or organizational practices, programs, or initiatives demonstrating empirical evidence of effectiveness [1]. There is significant scholarly evidence that EBIs can impact the well-being, support policy and decision-making, and improve quality of life, but only if they are successfully disseminated and implemented [2, 3]. Unfortunately, approximately 30–40% of patients do not receive care based on evidence [4] as EBIs do not diffuse or get taken up automatically or passively [5].

Implementation is often fraught with high failure rates that can lead to limited benefit, slow and haphazard change, underuse of effective evidence, poor return on investment, suboptimal outcomes, and significant opportunity costs [6–8]. The resulting implementation gap is a critical issue worldwide [9, 10], particularly for those who fail to receive evidence-based care [11]. Such challenges have recently been exacerbated by the unanticipated pivots to virtual delivery and rising population health needs resulting from the COVID-19 pandemic [12].

### The challenges of implementation

Innovation researchers and developers often focus on demonstrating efficacy and effectiveness and minimally consider how organizations may successfully implement them [13]. The ultimate impact of health innovations depends not only on their effectiveness but equally on their reach in the population and the extent to which they are implemented with high levels of fidelity [14]. Many organizations struggle with the ‘how to’ of effective EBI implementation. The ‘train and hope’ approach to practice change is ubiquitous but insufficient [15–17]. Selecting an EBI and training practitioners with little attention to contextual factors or exploring the organizational conditions necessary for effective delivery can render the effort unsuccessful. In one study, primary care clinics whose implementation plan was developed ‘on the fly’ noted double the time to implement an intervention (mean 623 days versus 314 days) versus the clinics that followed a pre-determined specific implementation plan [14]. In their review of how implementation impacts program outcomes, Durlak and DuPre [18] reported a significant positive relationship between the level of implementation monitoring and intervention outcomes for 76% of the studies. This link between implementation and program outcomes has been demonstrated in many other reviews [19–21]. One underlying problem is that organizations often have minimal knowledge or capacity to engage in evidence-informed implementation (i.e. they are not aware of the implementation science evidence, and when they are, they have difficulty applying it) [22–25].

Currently, implementation guidance is provided in static documentation or guides [e.g. [26–29]] that are non-interactive or non-adaptive for users, overly academic, and rarely evaluated. Intermediary organizations [30] that provide implementation facilitation and purveyors who develop, market, and support EBIs are often sector-specific (supports are not available to all users) and rely on costly in-person resources that are soft-funded; this creates limited capacity to support implementation at scale. Furthermore, organizations that support implementation struggle to keep pace with emerging implementation evidence and with communicating evidence in ways people can understand and apply [31, 32]. As a result, healthcare organizations lack clarity on implementation science methods and how to prepare and manage the change process. Implementing organizations require clear direction on what needs doing, how to do it, what factors prepare or hinder change, and a path forward illuminated by implementation science [33].

### The need for pragmatic guidance in implementation

The implementation gap gave rise to the field of implementation science—the scientific study of methods to promote the systematic uptake of research findings and other evidence-based practices [34]. While the interdisciplinary nature of implementation science is an asset to the field, many find it complex and challenging to apply in real-world settings [35–37]. We contend that existing guides and resources are static, hard to use, and fail to offer interactivity to support real-time implementation planning. Although implementation science seeks to reduce the research-to-practice gap, recent critiques suggest we may be recreating it. There exists a gap between scientific knowledge of implementation and its use in real-world implementation efforts [38]. Poor dissemination hampers the application of implementation knowledge in organizations.

The concept for the digital tool began with the development of a resource called The Implementation Game (TIG) [39]. TIG was designed to guide implementing organizations through implementation planning using a stepwise process that integrates implementation evidence, models, and frameworks. The resource invites users to develop an implementation plan using an approach that integrates and simplifies implementation evidence into five core elements: (i) implementation teams [40] (who lead the implementation work within the implementing organization), (ii) process [41] (four implementation phases and related activities), (iii) determinant factors [42, 43] (factors that hinder or support implementation in their context), (iv) strategies [44] (how barriers can be addressed), and implementation outcomes [45] (effects of deliberate actions to implement EBIs).

Launched in November 2019, 185 copies of TIG were disseminated to users in Canada, the United States and internationally. TIG’s usefulness was explored with an online survey, but the pandemic interrupted its in-person use in practice settings yielding a small sample (*n*=16). The survey queried the usefulness of the TIG worksheet for guiding process activities, TIG cards which conveyed more detailed information about concepts, and the TIG game board depicting the core implementation components. Feedback was positive and constructive. The worksheet helped guide users through the critical implementation phases, while the cards and board game seemed redundant. Additional input from 22 teams in virtual implementation workshops in 2022 revealed that users found the TIG helpful in planning, would recommend it, and were highly satisfied [46]. TIG feedback led to a new resource, The Implementation Roadmap (TIR), with a more detailed workbook supported by an 8 × 11-inch laminated poster highlighting the steps in the pathway and cross-cutting considerations for planning [47].

Preliminary work also included a planning meeting to work on persona development. The planning meeting supported the design stage to produce essential outputs for the build stage. First, we identified intended users and captured their needs, preferences, and implementation experiences to inform the design. We then developed personas over two consecutive virtual meeting days with target users in our network. Personas drive a collective understanding of how users will interact with the tool. Completing this essential user design step helped to ensure the tool would be useful and intuitive and support implementation by the intended users in a manner that meets their needs and preferences. Persona development focused on the following questions about users: who they are; their motivations to implement evidence; their intention to use The Implementation Playbook; the functionalities they would like to see in the tool; and how we could motivate, communicate, and support its use. A minimum requirements document was then shared with our design and development team, Pivot Design Group.

### The pivot to digital

The recent COVID-19 pandemic and the subsequent need for online digital resources revealed a window of opportunity to harness the paper-based TIR digitally to provide better ease of use and incorporate project management functionality [48–50]. The health benefits of digital tools are widely noted in the literature [51, 52], including user accessibility, learnability, navigation, control and input, and data collection [40] and are amenable to the integration of emerging evidence and tools over time [53]. Some e-health technologies have emerged in recent years to support implementation, but they are population or disease-specific. For example, The Implementation Pain Practice Change (ImPaC) Resource is an evidence-based online and interactive resource that guides healthcare teams through a practice change specific to improving infant pain assessment and management in the NICU [54]. Similarly, the ImpleMentAll project developed the ItFits-toolkit to support barrier and strategy tailoring and evaluated it with the implementation of internet-based Cognitive Behavioural Therapy (iCBT) [55]. Although eHealth technology has emerged as a solution for bringing evidence into practice, pragmatic and engaging implementation tools that guide and simplify the implementation process for all types of innovations in various organizations do not exist. Moreover, few resources address the organizational requirements for effective implementation [56, 57].

### The Implementation Playbook

This project aims to develop The Implementation Playbook (TIP), a digital tool that can systematically guide healthcare organizations through an intentional, explicit, structured, and evidence-informed approach to implementation. The proposed tool will incorporate the TIR’s core implementation elements coupled with interactive and dynamic online delivery to guide implementation over time and provide users with functionality to plan and monitor implementation. In addition, the electronic interface will be populated with automated links to other tools and resources and provide project management functionality to track implementation activities [58–62]. Upon completing the digital prototype or minimum viable product (MVP), we will test its feasibility by examining its use in six healthcare organizations. Feasibility studies aim to determine whether an innovation is feasible and appropriate for further development and more rigorous efficacy evaluation [63, 64].

## Methods

### Design and objectives

The study integrates implementation science, digital design and development, and health services research and employs a theory-driven multiple case study design [65] with convergent, explanatory mixed methods [66]. Quantitative data on the use of the tool will be integrated with qualitative data on how it was experienced, its usefulness, barriers and facilitators to its use, and desired features and functions for the next iteration. User-centred design principles (i.e. design that is concise, clear, and consistent and provides the user with autonomy) will guide the design and development of the digital tool [67]. We will then explore the tool’s feasibility for supporting EBI implementation in six organizations.

Development of the Playbook will occur in two phases: Phase 1: design, user testing, and development of an MVP, and Phase 2: feasibility testing of the MVP in six healthcare organizations (Table 1). We describe the specific objectives associated with each phase below.

**Table 1:** Organization sampling characteristics

Site	Organization type	Innovation delivery mode	Type of implementation support
1	Community Child & Youth Mental Health	In-person	Playbook alone
2	Adult Mental Health & Addictions	In-person	Playbook alone
3	Child, Youth and Adult Mental Health & Addictions	In-person	Playbook + facilitation
4	Health/Quaternary Care Hospital	In-person	Playbook alone
5	Health/Centre for Digital Therapeutics	eHealth technology	Playbook alone
6	Community Child & Youth Mental Health	eHealth technology	Playbook + facilitation

#### Phase 1: Development and usability testing of the Playbook

##### Objective 1: MVP co-design and development

Successful digital tool design requires a user-centred process from concept through to design, development, quality testing, implementation, and adoption, and frequently fails when established practices are not used [68]. eHealth technologies designed and developed based on assumptions about end-user motivations, goals and needs are often less effective than those that engage end-users throughout the process [68]. To optimize the relevance of the Playbook, we will employ a ‘user-centric’ approach in which end-users are central to the design process at each design phase and will allow for iterative modifications on content and functionality that meet user needs best. A ‘user-centric’ approach is paramount for user engagement with the tool and its effectiveness [68]. Collaborators, Pivot Design Group, were selected from three vendor bids to lead the design and development work.

The design phase will use discovery phase outputs on personas to sketch, ideate, visualize, and prototype the concept into life. First, we will outline the information architecture and sitemap from user personas, beginning with a series of task flows. Each task flow, or user flow, will be refined to outline the basic user experience and further flesh out the interaction design, from sketches through to wireframes, that outline the priority of information, content hierarchy and key content formats. Wireframes strategically filter the content in a format that considers how users interact with the content on the screen (no visual design, only black and white “blueprints” at this point). Next, we will create a mood board that captures the overall look and feel of the visual user interface and iterate through graphic layouts to come to a design that suits users’ priorities and contexts. All team members and collaborators will be involved and influence this process through discussion meetings guided by Pivot Design Group, which will seek end-user input on tool functionality, task flows, and visual display.

##### Objective 2: Usability testing

We will develop a click-through static prototype for one round of controlled usability testing to validate certain functions and task flows before designing the entire visual user interface; Pivot Design Group will lead this work. We will recruit 8–10 participants with varied gender perspectives and implementation experience to undergo a 45–60-min guided user testing process to test key features of the Playbook, including navigation and flow, functionalities (i.e. adding an activity or task), readability and accessibility. This number of participants allows for the saturation of trends across users with varied implementation experiences. We will recruit usability participants from our network via email, and their participation will be consented by Pivot Design Group, who will conduct the testing. Data will be collected for development purposes only and shared with the research team in aggregate. Questions asked will centre on accessibility and usability using a Think Aloud technique, where the participant verbalizes their thoughts and asks questions while they review the MVP [69]. Pivot Design Group will incorporate usability test results into a final round of wireframes and develop the final MVP for feasibility testing in Phase 2.

#### Phase 2: Feasibility testing of the Playbook

##### Sampling

The unit of analysis is the implementing organization. We sent an email invitation to six organizations in our network to test the feasibility of the Playbook by using it to implement an EBI of their choice from the start of implementation (see Table 1). Six organizations provide a suitable sample for achieving saturation in the check-in meetings [70]. We used maximum variation purposive sampling [71, 72], widely used in qualitative implementation research, to identify information-rich cases based on organization type (i.e. health, mental health, child/youth, adult) and two additional characteristics for context variability: EBI delivery mode (i.e. the EBI is delivered in-person or via eHealth technology); and type of implementation support (i.e. Playbook only, Playbook + purveyor or intermediary support). The type of implementation facilitation is an important context to test because it is a form of support used in practice. We imagine the Playbook could enhance how purveyors and intermediary organizations provide facilitation and create efficiencies for optimal implementation. We then solicited interest and participation from organizations that met these criteria within our network. The organizations we approached were known to the research team. The implementing organizations will form implementation teams to include ~3-5 staff with requisite skills to inform the implementation of the target EBI in their setting (e.g. knowledge of the EBI to be implemented, organizational workflows and clinical processes, and implementation process) [73]. We expect to engage with approximately 18–30 individuals in total.

##### Objective 1: Exploring current approaches to implementation

A baseline implementation survey will be shared for completion by the implementation team lead at each of the six implementing organizations to capture current approaches to implementation. In addition, a demographic survey administered to all participating implementation team members will collect demographic information on gender, age, implementation experience, and employment history. We will use REDCap electronic data capture tools hosted at Yale University [74, 75] to administer all measures and present data descriptively to depict team demographics and established implementation procedures across organizations.

##### Objective 2: Feasibility testing of the Playbook

*Target EBIs.* Before the Playbook launch, participating organizations will identify the EBI they have chosen to implement. The two intermediary organizations will identify the organizations and EBIs they will support and will be at liberty to support them as needed. We will intentionally provide minimal direction regarding the nature of the target EBIs since it is not yet known for what types of innovations it will be useful. We suspect that, at a minimum, EBIs must be complex enough (i.e. include multiple core components, not plug-and-play) to require a detailed implementation process. Multiple core components require explicit exploration of how they align with the implementing organization’s functions and structures. The target EBI must be supported by evidence and ready for implementation, and could be a practice, program, intervention, or initiative; delivered in person or via eHealth technology and targeted to adults or children.

*Access to the Playbook.* The implementation team lead at each of the six implementing organizations will be invited by email to access and register their project with the tool housed on a protected cloud-based server. All Playbook users will also receive a short (2-min) promotional video to engage, motivate and highlight Playbook functionalities and relative advantage. The video is not for training purposes since our premise is that built-in facilitation will be sufficient to enable self-directed use of the tool. All implementation leads will invite their team members to join their registered project space (e.g. create a login to interact with their team members on the tool). Two organizations in the Playbook + facilitation condition will share Playbook access with the intermediary or purveyor organization providing implementation support. Four sites in the Playbook-only condition will proceed without external implementation facilitation. All organizations can request technological assistance, and any requests for implementation facilitation from the Playbook-only sites will be addressed and documented in logged field notes. We will redirect technical issues and bugs to Pivot Design Group.

*Data collection.* Implementation is a varied and dynamic process, and measuring user experience in the moment is important. We selected 3-month check-in intervals to allow organizations to advance through implementation activities while balancing our need to monitor how the implementation is proceeding and minimize meeting burden. We will use the Microsoft Teams videoconference platform for check-in meetings with each implementation team, lasting approximately 60 min and conducted by MB and KP, both female investigators with doctoral training in psychology and health services research. Field notes captured in real-time using Microsoft Teams transcription and audio recording features will support rigour. This rapid analysis method is effective [76] and does not require costly and timely transcription. Once participant consents are secured, we will distribute the baseline implementation process survey for completion by the team lead in advance of the first check-in meeting. We will also distribute the demographic surveys for completion by each team member. These data will capture each organization’s prior implementation experience and approach. In addition, an adapted Organizational Readiness for Implementing Change questionnaire (ORIC) [77] will be administered to all implementation team members via REDCap during the baseline meeting to assess readiness to use the Playbook tool.

We will elicit how users are progressing with their implementation using the Playbook, which features are helpful, and any implementation needs not adequately addressed by the tool at quarterly check-in meetings. Probes [78] will identify usability issues in using the Playbook, including (1) description of the issue (i.e. how it fell short of meeting user needs and the consequences); (2) severity (i.e. how problematic the issue was ranging from 0 [“catastrophic or dangerous”] to 4 [“subtle problem”], adapted from Lyon et al. [79] and Dumas and Redish [80]; (3) scope (i.e. # of tasks affected by the issue); and (4) level of complexity (i.e. how simple the issue was to address [low, medium, high]). We will allow time at each check-in meeting for organizations to raise issues, ask questions and share comments. For the two organizations in the Playbook + facilitation condition, we will probe how they used support from the intermediary or purveyor organization. We will track emergent problems or queries with the tool via a built-in feedback button and analyse issue type, severity, and scope. Technical bugs will be addressed immediately by Pivot Design Group. Meeting transcripts will be shared with each implementation team and with intermediary organizations for comment or correction.

Implementation team members will also individually complete an adapted System Usability Scale questionnaire (SUS) [81] at each check-in meeting via REDCap. The SUS provides a reliable tool for measuring usability and consists of a 10-item questionnaire with five response options, from strongly agree to strongly disagree. SUS has become an industry standard because it is a straightforward scale to administer to participants, can be used on small sample sizes with reliable results, is valid and can effectively differentiate between usable and unusable systems [82, 83].

Quarterly meetings will also be held with the two implementation support organizations to learn how they integrate the tool into their facilitation process. Data captured in MS Team transcription will be coded for procedural changes, barriers and facilitators, and tool advantages and disadvantages.

Metrics from the Playbook content management software and Google Analytics will capture how users progressed through the tool’s steps and activities and how long they took to do so (time/efficiency). Metrics will include (1) *Duration*—time taken for completion of implementation phases (efficiency); (2) *Adherence* to the implementation steps and activities over time (i.e. did they complete Playbook activities and follow steps as intended as evidenced by user inputs within the tool); and (3) *Final Stage*—the furthest phase achieved in the implementation process. In addition, key implementation activities built into each implementation phase will provide milestone anchors for tracking user progression through implementation. Implementation cost-tracking will be added as a function in the following tool iteration (version 2.0).

The final month-24 check-in (or earlier, if implementation is attained) will involve two one-hour meetings per organization, scheduled within a month of each other. One meeting will follow the usual check-in protocol, and a second meeting will explore determinant factors that hindered or facilitated Playbook use; this will occur via team interviews informed by the updated Consolidated Framework for Implementation Research (CFIR2.0) [42, 84]. CFIR provides a taxonomy of operationally defined constructs associated with effective implementation, empirically derived from 19 theoretical frameworks, and organized into five domains: characteristics of the intervention (the Playbook), the inner setting, the outer setting, characteristics of individuals, and the process. The tool is adaptable for qualitative data collection (CFIRguide.com), and we will include all domains and factors. We will follow a modified rapid analysis (RA) approach that combines data collection and coding. The RA approach is an alternative to in-depth analysis of interview data that yields valid findings consistent with in-depth analysis, with the added advantage of being less resource-intensive and faster [76].

CFIR interviews will be conducted by two CFIR-trained research analysts with each implementation team using MS Teams’ transcription and audio-recording features. We will interview each organization’s implementation team as a group unless individual interviews are requested; this may occur if implementation teams include members with a varying role hierarchy, which may influence one’s intention to speak freely without fear of repercussion. Organizations will be reminded that the study focus is on the Playbook tool and its usefulness and feasibility rather than on their implementation performance. One analyst will facilitate the interview while a second analyst captures field notes directly onto a templated form that maps to CFIR domains and factors in the order presented in the interview protocol. CFIR has been extensively studied in various contexts [85–88], including the study of eHealth technology implementation [89]. In our experience, interviews with all 39 constructs can be conducted in 60 min [86–88]. Given limited evidence of constructs that may be more salient across contexts, we will include them all.

A final check-in meeting will also be conducted with the intermediary organizations to assess their overall experiences providing implementation facilitation alongside the Playbook. We intend to learn how the Playbook may be used as an adjunct tool to streamline their workflows and processes.

User input will include free-form content entered into the digital tool by the users as they work through the activities. For example, users are asked to discuss and describe how well the EBI fits with their current services, priorities, workflows, supports, community, and organizational values. User input at registration (first use) will include descriptive project details (i.e. target EBI, implementation timeline, funding, and team members). Links to resources and tools accessed by users will be tracked throughout. Back-end data will capture timestamped milestones and pathway progression as users work through the implementation phases and tasks.

##### Analysis

With a convergent design, we can integrate qualitative data (check-in notes, CFIR interviews, free-form user input) with quantitative data (tool metrics on use, ORIC, SUS) to develop a picture of the tool’s feasibility within different contexts. Both data types will be collected concurrently, apart from CFIR interviews, which we will administer at the end of implementation or 24 months. We will use visual joint display methods to depict user implementation experience with the tool [90]. Data integration will create a solid foundation for drawing conclusions about the tool’s usability, feasibility, and usefulness. In addition, this integration will lead to recommendations for improving the tool’s acceptability, feasibility, and effectiveness. Qualitative data analysis will allow us to explore user experience and tool functionality, how users progressed, implementation needs not adequately addressed, and barriers and facilitators to its use, which can inform subsequent revisions and user support before further testing. Reporting of qualitative results will follow the COREQ criteria [91].

*Qualitative*. Two research trainees will verify the fieldnotes from the ~48 check-in meetings (~8 per site over 24 months) collected in the MS Teams meeting transcripts and import data into MAXQDA 2022 [92]. The number and type of usability issues identified will be reported by organization and time point. The type of usability issue will be coded using a consensus coding approach and framework adapted by Lyon et al. [79] from cognitive walkthrough methods [93]. We will code issues associated with the user (i.e. the user has insufficient information to complete a task); hidden information (i.e. the user has insufficient information about what to do); sequencing or timing (i.e. difficulty with sequencing or time); feedback (i.e. unclear indications about what user is doing/needs to do); and cognitive or social (i.e. excessive demands placed on user’s cognitive resources or social interactions). Usability issue classification is critical because it facilitates data interpretation and provides more direct links between usability problems and Playbook redesign solutions.

Analysis of CFIR group interviews (*n*=8) will follow the modified RA approach [76]. Data captured on a templated summary table will be synthesized into summary memos by organization, including for the two intermediary organizations. Valence and strength will then be rated for each factor. The valence component of a rating (+/−) is determined by the influence the factor has on the process of using the tool to implement the innovation. The level of agreement among participants, the language, and the use of concrete examples determines rating strength (0, 1, 2). Two analysts are required for data collection and analysis: one conducts the interview, and the second takes notes in the CFIR data table during the interview. The interviewer reviews the coded template against the audiotape to ensure accuracy; they do not code independently of one another, but both analysts provide an independent valence rating and discuss differences to arrive at a consensus.

User free-form input will be captured per organization from the tool back-end and entered into MAXQDA software [92]. Two analysts will code these data independently with a coding tree aligned with the core elements (factors, strategies, process, equity considerations) and activities. Coding of emergent usability issues from these data will occur as above. Target EBI, initiating implementation context, team member demographics, and baseline implementation survey will be reported descriptively and inform data interpretation.

*Quantitative*. Ratings for both ORIC and SUS questionnaires use a 5-point Likert-type scale (1 = extremely disagree, 5 = extremely agree). They will be reported descriptively (range, mean, SD) by organization and usability issues (QUAL), adherence to core elements (QUANT), and final phase achieved (QUANT). SUS ratings will be analysed within organizations for changes across time intervals. Tool metrics will capture activity duration (dates of first and last activities completed within each phase to ascertain the number of implementation days), adherence (# and order of activities completed within a phase), and final phase achieved for each organization. These data will be explored against qualitative usability data between and within sites using joint display methods.

*Gender-based analysis*. Gender is important in decision-making, stakeholder engagement, communication, and preferences for EBI adoption [94]. Implementation may operate differently within and across genders under various circumstances [95] and requires decision-making that may shape what is implemented, how, and why. For example, leadership traits among leaders of different genders can influence the outcome of decision-making processes that are key to implementation. Gender may also affect how individuals use digital tools and eHealth innovations [96]. We will attempt to balance gender in the composition of our knowledge user group involved in tool development among usability testing participants and implementation teams. The analysis will be guided by a realist approach to discover what works, for whom, in what circumstances, and why. While we cannot control the gender composition within organization implementation teams, we will explore differences in our data.

*Limitations.* The Implementation Playbook has tremendous potential for impact due to its disruptive [97] capability (i.e. creating a resource or market where none existed), generic applicability and scalability. No existing technology does what the Playbook is designed to do.

Nevertheless, disruptive technologies bring inherent risks because they involve a new way of doing things. There is a risk that new technology can take years or fail to be adopted. Users of the Playbook may need help to follow all the steps and work through the activities, or they might prefer to implement with in-person external facilitation. Some organizations are more risk-averse and adopt an innovation only after seeing how it performs for others. Over time, we can leverage early adopters by highlighting the Playbook’s usability, feasibility, relative advantage, positive peer pressure and tension for change and by showcasing the experiences of champion users.

## Discussion

Poor implementation, regardless of intervention effectiveness, is costly and wasteful. To this end, we aim to produce a pragmatic solution that challenges the status quo in how organizations use or fail to use implementation science to inform their EBI implementation. This paper describes the protocol for a multi-phased research study to develop and test a digital tool to support the effective implementation of evidence-based innovations in various healthcare organizations and for various EBIs. We will produce a first-in-kind tool and learn whether it can feasibly be used to support implementation. We will identify what revisions may be needed and whether the tool can be used autonomously (without external facilitation), in different healthcare contexts, as an adjunct to external facilitation and with different EBIs. This work will inform the next iteration of the tool and preparation for an effectiveness study.

We hope to demonstrate that the Playbook enables self-directed implementation independent of costly external facilitation. We also intend for the tool to be universally useful in any healthcare context and for any type of EBI due to the universality of core elements from implementation science (i.e. implementation team, process, factors, strategies, and outcomes). The universality of these core implementation elements is analogous to the Plan Do Study Act model that guides quality improvement across contexts. The core elements stem from published work about process [98], determinant factors [42], strategies [44], implementation outcomes [45], and equity considerations [99] These core elements have guided our facilitation work in varying contexts (i.e. MB and JB have used the core components to guide implementation for >50 teams in international implementation initiatives). Tailoring these core elements to organizational context occurs in how users apply them in their planning and execution. The proposed tool is innovative and potentially disruptive; to our knowledge, there is no existing tool that integrates multiple implementation core components to facilitate EBI implementation across organizations and types of innovation.

## Data Availability

Not applicable.

## Abbreviations

CFIR: Consolidated Framework for Implementation Science
EBI: Evidence-based innovations
IS: Implementation Science
ORIC: Organizational Readiness for Implementing Change
SUS: System Usability Scale
TIG: The Implementation Game
TIR: The Implementation Roadmap
TIP: The Implementation Playbook
